# Botanical Origin of Galician Bee Pollen (Northwest Spain) for the Characterization of Phenolic Content and Antioxidant Activity

**DOI:** 10.3390/foods12020294

**Published:** 2023-01-08

**Authors:** Sergio Rojo, Olga Escuredo, María Shantal Rodríguez-Flores, María Carmen Seijo

**Affiliations:** Department of Vegetal Biology and Soil Sciences, Faculty of Sciences, University of Vigo, 32004 Ourense, Spain

**Keywords:** *Apis mellifera* L., bee pollen, botanical source, phenols, flavonoids, radical scavenging activity

## Abstract

Bee pollen is considered a natural product, relevant for its nutritional and antioxidant properties. Its composition varies widely depending on its botanical and geographical origins. In this study, the botanical characteristics of 31 bee pollen samples from Galicia (Northwest Spain) were analyzed; samples have not been studied until now from this geographical area. The study focused on the evaluation of the influence of plant origin on total phenol and flavonoid contents and antioxidant activity measured by radical scavenging methods. The multivariate statistical treatment showed the contribution of certain pollen types in the extract of bee pollen as to phenols, flavonoids and antioxidant capacity. Specifically, the bee pollen samples with higher presence of *Castanea*, *Erica*, *Lythrum* and *Campanula* type indicated higher total phenol and flavonoid contents and antioxidant activities according to the principal component analysis. On the contrary, *Plantago* and *Taraxacum officinale* type contributed a lower content of these compounds and radical scavenging activity. The cluster analysis classified the bee pollen samples into three groups with significant differences (*p* > 0.05) for the main pollen types, total phenol and flavonoid contents and antioxidant capacities. These results demonstrate the richness and botanical diversity in the pollen spectrum of bee pollen and enhance the possible beneficial nutraceutical properties of this beekeeping product.

## 1. Introduction

Plant pollination is accomplished by transferring pollen grains from the flower stamen of one plant to the stigma of another plant with agents such as wind, water, and insects [1,2,3]. Bees are actively involved in pollination by collecting and dispersing pollen from flower to flower. In addition to this essential function of ecosystem maintenance, the bees, during the collection of pollen, mix the grains with their own secretions, agglutinating it as small pellets on the hind legs of the insect, which are then transported to the hive. In beekeeping, these pellets generated by collecting pollen from flower stamens by the European honeybee *Apis mellifera* L. are known as bee pollen.

The pollen grains are recognized in apiculture as a source important of proteins, minerals and fats and are used mainly as food for the larvae and younger bees in the early stage of development inside the hive [1]. Moreover, since ancient times, pollen loads have been daily consumed throughout the world. Currently, due to both a trend towards natural diet supplementation and medical applications taking advantage of its numerous anticancer, anti-obesity, antimicrobial, anti-inflammatory and antioxidant properties, it is gaining more attention [4,5,6,7].

Bee pollen as a mixture of floral pollens collected by bees widely varies in shape, color, size, weight and in chemical composition. In the group of basic chemical substances, there are proteins, amino acids, lipids and fatty acids, carbohydrates, phenolic compounds and enzymes, as well as vitamins and bioelements [1,8,9]. Considering this excellent nutrient profile, bee pollen provides a significant daily intake of nutrients and complements the human diet. At the same time, this bee product comprises many compounds, especially rich in biologically active substances that differ according to the origin of the plant species visited by the bees. Therefore, in addition to its importance as a functional food, bee pollen is gaining special attention due to its active natural metabolites, especially derivatives of essential amino acids, polyphenolic substances, vitamins and lipids [4,6,9]. Although several natural metabolites interfere in free radical scavenging activity, it appears that phenolic acids and flavonoids are responsible for most of the antioxidant properties [4,5,10,11]. However, the deviations in the antioxidant activity and polyphenolic content between pollens are remarkable, as a consequence of the particularities of the plant species source and different geographical areas [3,12].

On the other hand, consumers increasingly demand quality, safe and healthy food. This is the consequence of a proper monitoring of traceability and physicochemical characterization of food. Even though bee pollen is known as a potent natural food, its physicochemical characteristics and nutritional composition are ambiguous and vary greatly depending on bee species, and the botanical and geographical origins [3,4,6]. Palynology analysis is the most widely used method worldwide for identifying the botanical origins of bee products, as well bee pollen [4]. However, research studies that include a detailed pollen profile of this type of matrix and its relationship with the nutritional composition are infrequent [2,13,14,15].

In recent years, bee pollen as a commercial product has gained significant profitability in the beekeeping sector, taking advantage of its several functional properties as a health ingredient of multiples food [7]. Spain is the leading producer of bee pollen in the European Union. The unique qualities that contribute to beekeeping products are derived from the diversity in the flora, climate and soil of the Spanish territory. However, research on bee pollen from Spain is scarce from the point of view of its physicochemical and botanical characteristics, in comparison with other bee products such the honey. Polyphenolic and flavonoid compounds [16,17,18], in addition to profiling of amino acids, sugars, alkaloids and nucleic acids [18], and the amino acid content [19] of bee pollen collected in Spain were reported.

In order to guarantee well-characterized products, in recent years geographical indication has been included in the commercialization of bee products as an essential tool. Thus, the evaluation of the quality parameters and nutritional compounds of bee pollen from different geographical areas could provide differentiating information for producers in the sector and consumers [8]. Specifically, in the Northwest of Spain (Galicia) there are several unifloral honeys characterized and protected at European level by the quality scheme of Protected Geographical Indication (PGI) *Miel de Galicia* [20]. In this context, this study contributes to the knowledge of the bee pollen’s characteristics, provides information for the inclusion as a quality product and consequently, favors the diversification of bee products.

Due to the variability in the chemical composition of bee products related to botanical origin, a set of bee pollen samples produced in different locations of Galicia was analyzed. The aim of this study was to evaluate the botanical diversity of bee pollen samples from Northwest Spain by means of palynological analysis and their influence on the total phenol content (TPC), total flavonoid content (TFC) and radical scavenging activity (DPPH and ABTS).

## 2. Materials and Methods 

### 2.1. Bee Pollen Samples

**Figure 1:**
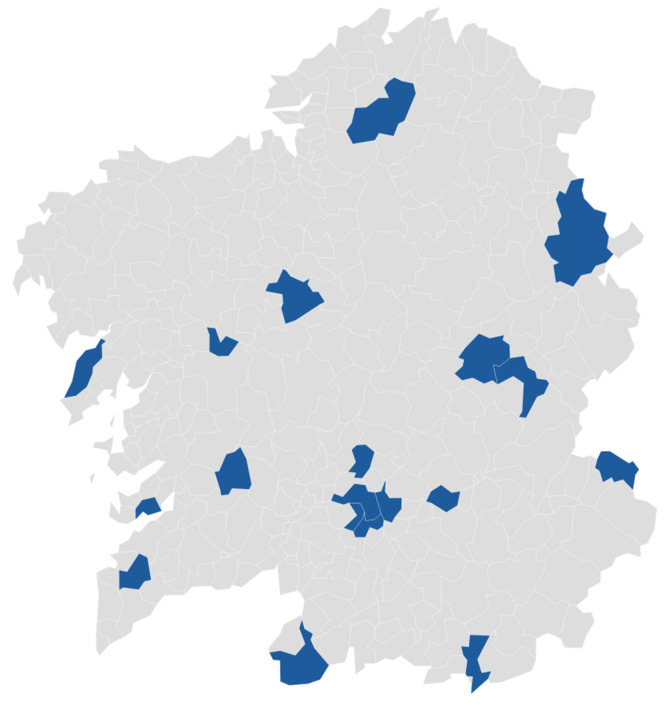
Distribution of geographical origin by municipality in blue of the bee pollen samples in Galicia (Northwest Spain). Created with Datawrapper.

In this study, 31 bee pollen samples from Galicia (Northwest Spain) were analyzed. The samples were collected using pollen traps from *Apis mellifera* hives in 19 different apiaries. The geographical origin of the pollen samples collected is shown in Figure 1. The apiaries were distributed in the four provinces of the Galician autonomous community (A Coruña, Lugo, Pontevedra and Ourense). Specifically, in A Coruña the samples were collected from 4 apiaries (9 samples), in Lugo from 3 apiaries (4 samples), in Pontevedra from 3 apiaries (4 samples) and in Ourense from 9 apiaries (14 samples). After collection, the bee pollen samples were cleaned of impurities (such as parts of dead bees or remains of wax), and until further analysis were stored at −20 °C.

### 2.2. Determination of Botanical Origin

Botanical origin was determined using a melissopalynological procedure. First, the original samples were conveniently homogenized, then 1 g was weighed and placed in separate vials. Subsequently, a colorimetric separation was carried out to obtain different subsamples, which were weighted and dissolved in distilled water (15 mL). The solutions were shaken for 10 min and at 4500 rpm for 5 min centrifuged. An aliquot of 100 μL was taken from the sediment to prepare the slides. An optical microscope (Nikon Optiphot II, UK Ltd., London, UK) was used to identify the botanical origin of the different subsamples. The pollen spectra of the samples were determined considering the weight of each subsample and its botanical origin. The results were expressed in percentages.

### 2.3. Preparation of Bee Pollen Extracts

The extracts of the bee pollen samples were prepared according to the method of Gabriele et al. [21], with minor modifications. 0.5 g of each pollen sample was dissolved with 80% ethanol to a concentration of 0.01 g/mL. These extracts were gently shaken in the dark for 5 h and subsequently macerated for 24 h. After this time, the extracts were centrifuged for 10 min at 4500 rpm, and subsequently properly stored in amber-colored glass containers to avoid direct incidence of light.

### 2.4. Assessment of Total Phenol Content

The determination of the total phenolic content (TPC) was carried out based on the method developed by Singleton and Rossi [22] adapted to bee pollen. 1 mL of the bee pollen extract solution (0.01 g/mL) was dissolved with 1 mL of Folin-Ciocalteu reagent and 10 mL of distilled water. After gently stirring and standing for 2 min, 4 mL of 7% Na_2_CO_3_ solution was added and made up to 25 mL with distilled water. The solutions were measured using a UV–Vis spectrophotometer (Jenway 6305, Fisher Scientific, Loughborough, UK) at an absorbance of 765 nm after being kept in the dark for 1 h. Gallic acid solutions were used to obtain the calibration curve. The results of TPC as gallic acid in mg/100 g were expressed.

### 2.5. Assessment of Total Flavonoid Content

The total flavonoid content (TFC) was measured using the method of Arvouet-Grand et al. [23] adapted for pollen. An aluminum chloride solution is used for its reaction with the flavonoid compounds present in the bee pollen solutions. Thus, 2 mL of the bee pollen extract solution (0.01 g/mL) was dissolved with 0.5 mL of 5% aluminum chloride and distilled water to final volume of 25 mL. After 30 min in the dark, the prepared solutions turned yellow, at which time the absorbance at 425 nm with UV–Vis spectrophotometer was measured (Jenway 6305, Fisher Scientific, Loughborough, UK). Quercetin solutions were used as reference pattern and the results of TFC were calculated as quercetin in mg/100 g.

### 2.6. Assessment of Antioxidant Activity by Radical Scavenging Assay: DPPH and ABTS

The radical scavenging capacity of the bee pollen extracts was determined based on the scavenging ability of the antioxidants towards the stable 2, 2-diphenyl-1-picrylhydrazyl radical known as DPPH method [24]. The scavenging activity on pollen extracts, mixed with 2.7 mL of a DPPH solution (6 × 10^−5^ M) was measured. The pollen sample solution and the blank-DPPH solution were incubated at room temperature for 30 min in the dark. The absorbance with a UV–vis spectrophotometer was measured at 517 nm.

The radical scavenging activity by ABTS assay was determined according to a method reported by Re et al. [25]. The ABTS solution was prepared by reacting ABTS 7 mM in water with 2.45 mM potassium persulfate. ABTS stock solution was left at room temperature for 12–16 h in the dark until it reached a stable oxidative state. ABTS stock solution was prepared by dilution with ethanol to give an absorbance of 0.70 ± 0.02 at 734 nm. Then, 980 μL of this solution was mixed with 20 μL of the ethanolic extract of bee pollen sample and finally, the absorbance was measured at 734 nm.

The antioxidant activity of bee pollen samples was expressed as percentage of DPPH and ABTS calculated using the following equation: Scavenging activity (%) = [(AbsB−AbsS)/AbsB] × 100, where AbsB is the absorbance of the blank solution according to radical used and AbsS is the absorbance of the pollen extract solution.

### 2.7. Data Analyses

The significant differences between the pollen types identified by palynological analysis, TPC, TFC and antioxidant variables of bee pollen samples set were determined using a Student’s *t*-test. The level of statistical significance was taken into account given a *p*-value (*p*) less than 0.05. Principal component analysis (PCA) was applied with the objective of providing a reduced interpretation of the variance of the data of the studied variables (main pollen types, TPC, TFC and antioxidant activity) in the bee pollen samples. With the aim to provide a simplified interpretation of the variance of the data set of the main analyzed variables (main pollen types, TPC, TFC and antioxidant activity), a principal component analysis (PCA) was applied. The data matrix was reduced to a small number of principal components to analyze the significant relationships between the variables. At the same time, groups of pollen samples were established using multivariate cluster analysis. This statistical approach grouped samples based on a data set of variables from cases with similar characteristics. Differences between the groups were tested using the Bonferroni test through post hoc comparison (*p* < 0.05). STATGRAPHICS Centurion XVI software (Statpoint Technologies, Inc., Warrenton, VA, USA) was used for treatment of data.

## 3. Results

### 3.1. Botanical Preference for Bee Pollen Production

**Figure 2:**
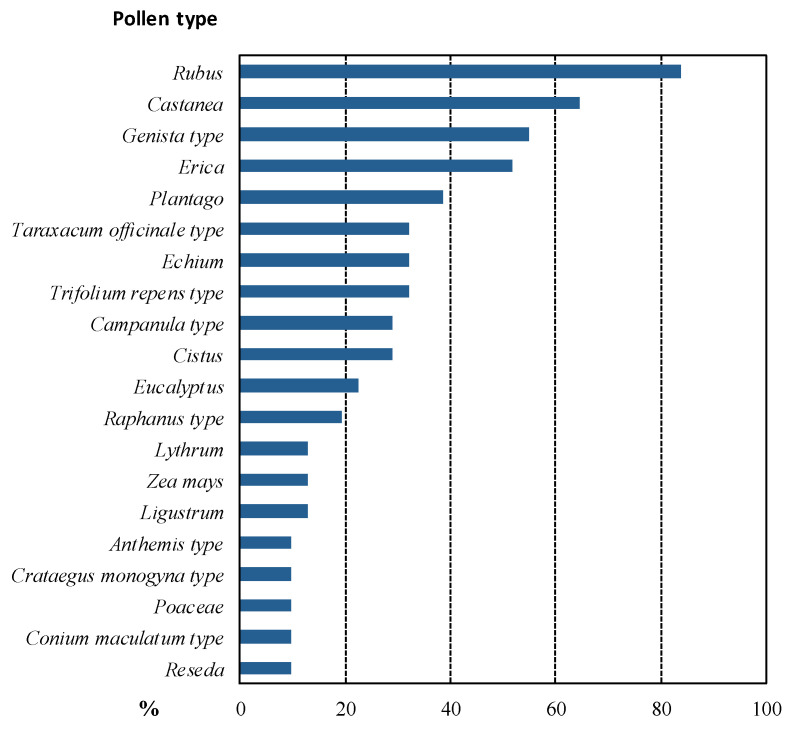
Frequency of the pollen types identified in the bee pollen samples.

The results of palynological analysis of the studied bee pollen samples are summarized in Figure 2 and Table 1. In the set of samples analyzed were identified fifty-one pollen types belonging to 32 families.

Considering the distribution of the different pollen types in samples, pollen grains from *Rubus*, *Castanea*, *Genista* type and *Erica* were present in more than 50% of the samples (Figure 2). Other frequent pollen types were from *Taraxacum officinale* type, *Echium* and *Trifolium repens* type (found in 30% of the samples). With regards the abundance of pollen types in each sample, some of the well-distributed pollen types such as *Rubus*, *Castanea*, *Genista* type, *Taraxacum officinale* type and *Lythrum* were found, at least in one sample, as dominant pollen (>45%) (Table 1). However, *Rubus* and *Castanea* were the most representative pollen types (26 and 20 samples, respectively), and those with the highest percentage counted in the pollen spectrum, with mean values above 22% and maximum value of more than 90% (Table 1).

### 3.2. Concentration of Total Phenol, Flavonoid and Antioxidant Capacity of Bee Pollen

The mean content of TPC and TFC was 1612.6 mg/100 g and 256.8 mg/100 g, respectively (Table 2). The range for TPC was between 771.8 mg/100 g and 2638.9 mg/100 g and for TFC between 90.8 mg/100 g and 639.3 mg/100 g. The antioxidant activity expressed as DPPH and ABTS had a mean value of 65.7% and 57.4%, respectively. The maximum value of DPPH found in the samples was 88.2% and for ABTS, of 79.3%. Significant differences were found between the mean values for all the variables analyzed in the bee pollen samples (*p* < 0.05).

### 3.3. Contribution of the Botanical Origin to the Phenolic Content and Antioxidant Activity of Pollen

The relationships between the botanical origin of the bee pollens and the polyphenol and flavonoid contents and the antioxidant capacity have been evaluated using a PCA. This multivariate technique reduced the dataset and revealed a six-component model with 81.47% of the variance of the data (Table 3). The first three components explained 53.40% of the data variability. The variables with higher weight in the first component were *Taraxacum officinale* type, *Plantago*, DPPH, TPC and *Castanea* (with coefficients above 0.30). In the second component, the higher coefficients corresponded to *Erica*, TFC and *Campanula* type (above 0.35), while the third component was related with the higher coefficients of TFC, DPPH, *Echium*, *Trifolium repens* type and *Rubus* (coefficients above 0.26).

**Figure 3:**
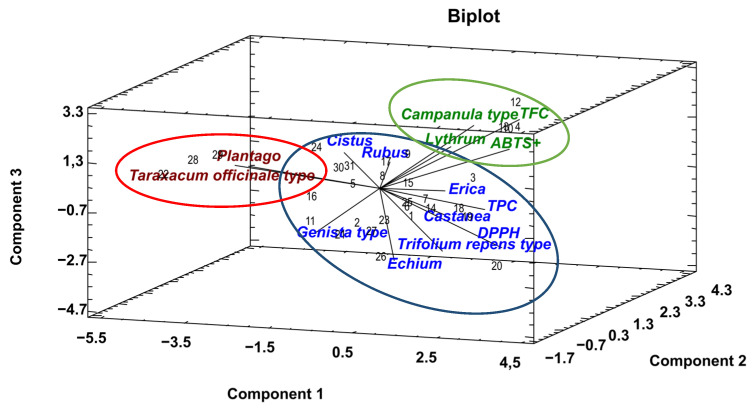
Plot of the first three principal components with the palynological and chemical variables obtained by PCA.

The projection of the relationships among the palynological and chemical variables on the three first components is shown in Figure 3. The graphic representation shows the close relationship of the pollen variables *Erica*, *Castanea*, *Trifolium repens* type and *Echium* with TPC and DPPH, whereas *Campanula* type and *Lythrum* are closely related to ABTS and TFC. On the contrary, *Genista* type, *Plantago* and *Taraxacum officinale* type had an inverse relationship. Therefore, the bee pollen samples with higher presence of *Erica*, *Castanea*, *Echium* and *Trifolium repens* type had higher TPC and antioxidant activity. Bee pollen with high presence of *Campanula* pollen type and *Lythrum* were characterized by higher concentration of TFC and antioxidant content.

**Figure 4:**
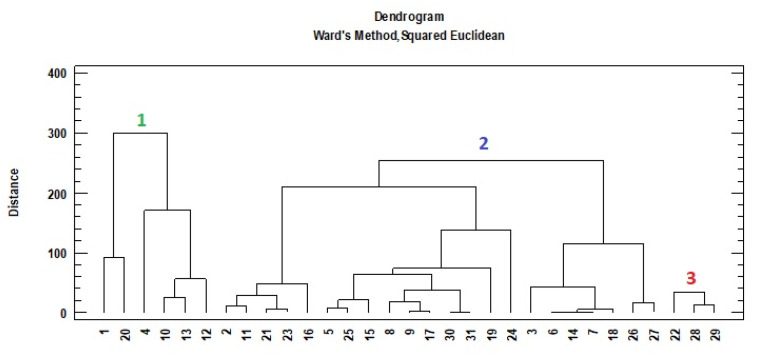
Dendrogram of cluster analysis with the distribution of bee pollen samples (1: group 1; 2: group 2; 3: group 3).

The cluster analysis was carried out with the palynological variables of greater representation in the pollen spectra, TPC, TFC and antioxidant activity (DPPH and ABTS). The results of this multifactorial analysis classified the pollen samples into three groups (Figure 4).

The first group (1) included six pollen samples with significantly higher percentages in *Erica* (20.1%), *Campanula* type (14.5%), *Lythrum* (11.5%), significantly higher in TFC (454.1 mg/100 g) in comparison to the other two groups (two and three), and DPPH (69.3%) respect to group three (21.0%) (Table 4). In group two, there is the largest number of pollen samples (22) and it was characterized by significantly higher mean percentage in the *Genista* type (12.1%), *Castanea* (28.4%) and *Rubus* (37.7%) pollen types, TPC (1741.4 mg/100 g), TFC (219.2 mg/100 g) and antioxidant activity (70.8%) compared to group three (21.0%). Finally, group three (with three bee pollen samples) included the samples with the significantly lower mean values in TPC, TFC and DPPH, but significantly higher proportions of the pollen types *Taraxacum officinale* type (50.2%) and *Plantago* (24.3%).

## 4. Discussion

The trend in the human diet is to consume foods with a high nutritional value, replacing the more conventional foods, as well as using them as supplements to provide one’s diet the energy and essential nutrients required for proper mental and physical development [26]. In terms of human nutrition, bee pollen is considered a natural substance that constitutes a potential source of compounds with diverse nutritional and antioxidant relevance. Since pollen comes from different plant species, the evaluation of quality, safety and its characterization depend on the botanical and geographical origins. Hence the importance of their characterization and differentiation [1,15,27,28]. According to Campos et al. [29] the correct control of the processing procedures declares a consistent composition and could be considered as an indicator of outstanding quality and properties of food products. Some countries are establishing internal regulations with the intention of favoring the quality control of this product. In addition, local growth and the potential for the sale of bee pollen through exports has motivated the creation of international regulations to standardize the analytical methods for the physical-chemical and nutritional analysis of bee pollen [1].

The beneficial functions of phenolic compounds for human health have been demonstrated by reducing oxidative stress and inhibiting macromolecular oxidation; they positively collaborate in reducing the risk of degenerative diseases [30]. Within the phenolic compounds, the flavonoids in the bee pollen matrix have been recognized as quality factors in terms of antioxidant capacity [3,29]. However, phenolic composition of bee products is conditioned by their botanical origin, hence the need to evaluate the particular botanical characteristics of bee pollen based on geographical origin. In the present study, the bee pollen samples with higher presence of pollen types from *Erica*, *Castanea*, *Trifolium repens* type and *Echium* had the highest TPC and RSA expressed in DPPH. The bee pollen with the highest TFC had the highest antioxidant capacity expressed as ABTS, coinciding with the pollen types with the highest presence of *Campanula* type and *Lythrum*.

The Galician territory is characterized by a transition zone composed of different types of climate, resulting an environmental diversity that favors the abundance of plant resources for bees. The plant species of greatest beekeeping interest in Northwest Spain are from the families Fagaceae, Rosaceae, Fabaceae and Ericaceae. *Castanea* and *Rubus* taxa (Fagaceae and Rosaceae, respectively) produce a high quantity of nectar and pollen during the flowering stage (between May and July in the lowest lands and the mountains, respectively), with important productions of unifloral honeys of this botanical origin [31,32]. Bramble and blackberry plants are the most common of the *Rubus* genus (Rosaceae), with almost 300 species growing in Central European. *Rubus ulmifolius*, *R. caesius*, *R. sampaianus*, *R. praecox* or *R. henriquesii*, among others, are the most widespread in Galicia [33,34]. *Castanea* trees produce a great amount of nectar and pollen, and are considered one of the best beekeeping resources in this geographical area, given the important production of honeys of this botanical origin [32]. Species of the family Ericaceae, and other taxa of Fabaceae, mainly *Trifolium repens* and *Genista* type, constitute the typical scrub of the area, with important beekeeping interest [35]. Undoubtedly, the botanical richness that characterizes the Galician territory confers the chemical particularities of bee products.

The TFC quantified in bee pollen from Northwest Spain was similar to that found in samples from locations in southern Spain [17] and Portugal [7,30,36]. Pascoal et al. [15] reported a higher TPC in bee pollen produced in Portugal (Northeast area), with a dominant abundance of pollen types *Erica*, *Echium* and *Castanea*. The abundance of these pollen types probably increased the phenol content in the set of bee pollen samples, coinciding with the significant relationship found in the PCA of this research. *Erica*, *Castanea* and *Echium* turned out to be the plant variables with weight in PCA, coinciding with the variables close to TPC (Figure 3). Mârghitas et al. [37] reported similar TPC in honeybee-collected pollen pellets from Romania separated by colorimetry (*Crataegus monogyna*, *Centaurea cyanus*, *Salix*), but TFC was higher than the bee pollen analyzed in this study.

On the other hand, bee pollen produced in the Sonoran Desert (North of Tucson, USA) with a pollen spectra characterized by the presence of *Prosopis*, *Yucca*, *Washingtonia*, *Larrea*, *Mimosa* and Chenopodiaceae had higher TPC [2] compared to the pollen samples of the present study. Other researchers also reported higher TPC in bee pollen from Brazil, with a dominant pollen representation of *Cecropia*, *Eucalyptus*, *Mimosa pudica*, *Elaeis*, *Eupatorium* and *Scoparia* [10], and with dominant pollen *Cocos nucifera*, *Miconia*, *Spondias* and *Eucalyptus* [13]. Kostic et al. [28] characterized sunflower bee pollen (*Helianthus annuus*) with *Taraxacum officinale* as accompanying pollen from Serbia by palynological analysis, with lower TPC and TFC than bee pollen from Galicia. *Taraxacum officinale* was also found in the pollen spectrum of studied bee pollen in our study, coinciding with the samples of lower TPC and TFC (Figure 3).

There are several antioxidant compounds involved in the oxidation of the bee products, and the total antioxidant activity is the most accurate measurement [38]. The objective of the determination of the antioxidant activity of bee pollen is to analyze the generation of free radicals due to the disappearance of antioxidants. The DPPH and ABTS methods are the most used and most stable to evaluate the antioxidant capacity of hydrogen-donating antioxidants (aqueous radical scavengers) and chain-breaking antioxidants (lipid peroxyl radical scavengers), although they also show differences [38]. The positive relationship between TFC and antioxidant activity of the bee pollen samples was referenced [10]. The abundance of specific species identified in the extract of pollen samples with high radical scavenging activity and TPC was related, such as *Sinapis alba*, *Robinia pseudoacacia* [12], *Salix alba* [37] or *Mimosa* [2]. In our study, the abundance of *Castanea*, *Erica*, *Rubus*, *Campanula* type and *Genista* type differentiated the bee pollen samples with higher radical scavenging activity, highly related to TPC and TFC (Figure 4 and Table 4). Bee pollen of *Taraxacum officinale* from Romania [37] and Poland [12] despite high TPC showed low antioxidant capacity by DPPH assay. Several researchers have concluded that the antioxidant capacities are not clearly associated with its total phenolic content [12,30,37]. It is possible that the presence of particular phenolic compounds determines the increase in antioxidant activity [13]. The polyphenolic profile is variable in bee pollen, and the antioxidant activity of polyphenols depends on the number and location of the hydroxyl groups it contains in its chemical structure [38]. Its chemical structure is conducive to scavenging free radicals, because the hydrogen atom from the aromatic hydroxyl group readily donates to the radical species and the stability of the quinone structure it turned out to support an unpaired electron [39]. This strong association is mainly attributed to flavonoids and cinnamic acid derivatives [3,12]. Hence, the importance of relating the botanical origin with the individual phenolic compounds, because they can contribute to the discrimination of the antioxidant capacity of some pollen samples based on their floral origin.

It has also been documented that differences in environmental conditions, soil or plant physiology may interfere in the free radical reactions and the ability to remove reactive oxygen species in this bee product [3]. Some authors supported the close relationship between the antioxidant capacity and the collection period of bee pollen, highlighting a higher antioxidant activity in bee pollen produced in a period of more UV-intense, specifically from the beginning to the end of summer [2]. Therefore, in addition to correct management practices, the time of collection of the bee product will influence the chemical and functional characteristics (closely linked to the flowering period) and must be taken into account by the beekeeper.

## 5. Conclusions

The botanical characterization of bee pollen is essential for the particular identification of its chemical composition. The results of multivariate statistical treatment applied to the bee pollen sample set revealed the influence of botanical origin on TPC, TFC and antioxidant capacity. *Castanea*, *Erica*, *Lythrum* and *Campanula* type have been characterized as the pollen types with the greatest influence on TPC, TFC and antioxidant activity (as indicated by the first two main components of PCA) of bee pollen produced in the Northwest of Spain. On the contrary, *Plantago* and *Taraxacum officinale* type contributed a lower content of these compounds in this geographical territory. These results provide some evidence for the healthy potential in which the free radicals of bee pollen are involved, promoting the consumption of this traditional food. Expanding these results with a larger number of samples of this botanical origin will help to confirm these conclusions.

## Figures and Tables

**Table 1:** Most-representative families and pollen types in the bee pollen samples produced in Galicia.

Family	Pollen Type	N	Mean	Standard Deviation	Maximum
Rosaceae	*Rubus*	26	29.3 *	27.4	94.5
	*Crataegus monogyna* type	3	0.5	2.1	11.7
Fagaceae	*Castanea*	20	22.6 *	27.7	91.1
	*Quercus*	2	1.8	7.9	42.1
Fabaceae	*Genista* type	17	9.8 *	19.0	61.2
	*Trifolium repens* type	10	2.6	7.7	39.5
Ericaceae	*Erica*	16	5.4 *	10.5	44.8
	*Calluna vulgaris*	2	0.6	2.3	11.2
Plantaginaceae	*Plantago*	12	2.9 *	7.3	27.0
Asteraceae	*Taraxacum officinale* type	10	5.9 *	15.7	60.3
	*Anthemis* type	3	1.0	4.7	26.3
Boraginaceae	*Echium*	10	2.6 *	5.7	22.2
Campanulaceae	*Campanula* type	9	3.1	8.8	37.5
Cistaceae	*Cistus*	9	1.8	5.3	28.2
	*Cistus psilosepalus*	2	0.2	0.7	3.6
Myrtaceae	*Eucalyptus*	7	0.7	1.8	7.8
Brassicaceae	*Raphanus* type	6	1.7	4.5	18.5
	*Brassica*	2	0.4	1.9	10.3
Lythraceae	*Lythrum*	4	2.2	9.8	54.5
Oleaceae	*Ligustrum*	4	0.5	1.6	7.3
Poaceae	*Zea mays*	4	0.3	0.9	3.3
	*Poaceae*	3	0.3	1.2	6.6
Apiaceae	*Conium maculatum* type	3	0.4	1.7	9.3
	*Foeniculum vulgare* type	2	0.5	2.9	16.0
Resedaceae	*Reseda*	3	0.1	0.3	1.5
	*Sesamoides*	2	0.5	1.9	10.2
Chenopodiaceae	*Chenopodiaceae*	2	0.2	1.0	5.4

N = number of pollen samples containing it. * Significant differences according to Student’s *t*-test (*p* < 0.05).

**Table 2:** Descriptive analysis of TPC, TFC and antioxidant activity expressed as DPPH and ABTS.

	Mean	Standard Deviation	Minimum	Maximum
TPC (mg/100 g)	1612.6 *	531.0	771.8	2638.9
TFC (mg/100 g)	256.8 *	150.0	90.8	639.3
DPPH (%)	65.7 *	20.5	17.0	88.2
ABTS (%)	57.4 *	12.6	32.8	79.3

* Significant differences according to Student’s *t*-test (*p* < 0.05).

**Table 3:** Number of components extracted and component weights for each variable included in PCA.

Components	1	2	3	4	5	6
Eigenvalue	3.68	2.32	2.01	1.73	1.37	1.11
Variance (%)	24.55	15.45	13.40	11.52	9.13	7.41
Variance cumulative (%)	24.55	40.01	53.40	64.93	74.06	81.47
Component weights						
TPC	0.40	−0.19	−0.01	−0.38	−0.06	0.07
TFC	0.22	0.46	0.27	−0.03	−0.09	0.24
DPPH	0.41	−0.10	−0.27	0.11	0.05	0.08
ABTS	0.29	0.23	0.19	0.02	0.01	−0.43
*Taraxacum officinale* type	−0.45	0.03	0.08	−0.22	−0.23	−0.08
*Echium*	0.00	0.10	−0.48	0.00	−0.46	0.11
*Campanula* type	0.12	0.38	0.25	−0.04	0.12	−0.30
*Cistus*	−0.06	−0.12	0.26	0.40	−0.24	0.23
*Erica*	−0.01	0.46	−0.20	0.10	0.20	−0.16
*Genista* type	−0.13	−0.15	−0.24	0.04	0.69	0.13
*Trifolium repens* type	0.06	0.30	−0.50	0.20	−0.22	−0.03
*Castanea*	0.31	−0.26	−0.02	−0.44	−0.17	−0.21
*Lythrum*	0.09	0.28	0.16	−0.26	0.04	0.70
*Plantago*	−0.43	0.08	0.05	−0.29	−0.15	−0.10
*Rubus*	0.13	−0.23	0.27	0.49	−0.18	−0.04

**Table 4:** Groups of bee pollen samples of each class obtained by the cluster analysis.

Groups	1	2	3
N (%)	6 (19.3)	22 (71.0)	3 (9.7)
TPC (mg/100 g)	1527.5	1741.4 a	838.3 a
TFC (mg/100 g)	454.1 a b	219.2 b	137.2 a
DPPH (%)	69.3 a	70.8 b	21.0 a b
ABTS (%)	65.0	56.9	46.2
Pollen types (%)			
*Taraxacum officinale* type	0.5 a	1.3 b	50.2 a b
*Echium*	4.0	2.2	3.1
*Campanula* type	14.5 a b	0.5 a	0.0 b
*Cistus*	0.0	2.5	0.0
*Erica*	20.1 a b	1.7 a	2.6 b
*Genista* type	2.2	12.1	8.6
*Trifolium repens* type	9.0	1.2	0.0
*Castanea*	12.5	28.4	0.0
*Lythrum*	11.5 a b	0.0 a	0.0 b
*Plantago*	1.5 a	0.4 b	24.3 a b
*Rubus*	13.3	37.7 a	0.0 a

N: number of samples. Same letter shows the significant differences between groups by Bonferroni test (*p* < 0.05).

## Data Availability

The datasets that were generated during and/or analyzed during the current study are available from the corresponding author on reasonable request.
